# Short-Term Effects of Balance Training with Stroboscopic Vision for Patients with Chronic Ankle Instability: A Single-Blinded Randomized Controlled Trial

**DOI:** 10.3390/ijerph18105364

**Published:** 2021-05-18

**Authors:** Kyung-Min Kim, María D. Estudillo-Martínez, Yolanda Castellote-Caballero, Alejandro Estepa-Gallego, David Cruz-Díaz

**Affiliations:** 1 Department of Kinesiology and Sport Sciences, University of Miami, Coral Gables, FL 33146, USA; kmk154@miami.edu; 2 Department of Sport Science, College of Sport Science, Sungkyunkwan University, Suwon-si 16419, Korea; 3 Department of Statistics, Faculty of Experimental Sciences, University of Jaén, E-23071 Jaén, Spain; mdestudi@ujaen.es; 4 Department of Health Sciences, Faculty of Health Sciences, University of Jaén, E-23071 Jaén, Spain; alejandroestepafisio@gmail.com (A.E.-G.); dcruz@ujaen.es (D.C.-D.)

**Keywords:** chronic ankle instability, balance training, stroboscopic vision

## Abstract

Chronic Ankle Instability (CAI) is one of the most common musculoskeletal dysfunctions. Stroboscopic vision (SV) training has been deemed to enhance somatosensorial pathways in this population group; nevertheless, until recently no studies have addressed the additional effects of this treatment option to the traditional therapeutic approach. Methods: To evaluate the effectiveness of a partial visual deprivation training protocol in patients with CAI, a randomized controlled trial was carried out. Patients with CAI (*n* = 73) were randomized into either a balance training, SV training, or a control (no training) group. For participants assigned into training groups, they received 18 training sessions over 6 weeks. The primary outcome was dynamic balance as measured by the Star Excursion Balance Test assessed at baseline and after 6 weeks of intervention. Secondary outcome measures included ankle dorsiflexion range of motion, self-reported instability feeling, and ankle functional status. Results: Better scores in stroboscopic training and balance training groups in all outcome measures were observed in comparison with the control group with moderate to large effect sizes. Stroboscopic training was more effective than neuromuscular training in self-reported instability feeling (cohen’s d = 0.71; *p* = 0.042) and anterior reach distance of the star excursion balance test (cohen’s d = 1.23; *p* = 0.001). Conclusions: Preliminary findings from the effects of SV Stroboscopic training in patients with CAI, suggest that SV may be beneficial in CAI rehabilitation.

## 1. Introduction

Ankle inversion sprain is one of the most common musculoskeletal injuries associated with sports practice [1,2]. The principal mechanism of injury consists of a sudden inversion movement in the ankle joint which can affect the ankle joint structures as well as the somatosensory system [3]. This may lead to residual symptoms after the first ankle sprain episode including recurrent episodes of giving way, chronic pain and swelling, subjective instability feeling and is associated with altered arthrokinematics, reduced ankle dorsiflexion range of motion (DFROM), and sensorimotor deficits which have been termed as chronic ankle instability (CAI) [4].

It has been reported that patients with CAI present altered postural control, muscle activation ratio, and impaired proprioception [5,6]. The management of postural control has been focused on the improvement of motor function by different strategies such as strength training, neuromuscular training, proprioceptive training, or a combination of all of these [7,8,9]. The reduction in somatosensory utilization in those with CAI due to the altered input of the ankle joint and foot complex requires intervention for the sensory receptors’ restoration [10,11].

Balance training is related to the improvement of postural control in those with CAI and has been deemed as an effective therapeutic approach for this population [7,8,9]. Taking into account the complex etiology of CAI, injury-related impairments interventions have been successfully applied to improve postural control, DFROM, or muscle strength [7,8,9]. However, it has been observed that focused intervention also provides improvements in other contributing factors of CAI beyond those that have been particularly addressed [12]. It suggested that a multimodal balance training including challenging tasks in multiple plains of space, displacements, and static and dynamic tasks during typical sports situations may play an important role in the prevention of ankle sprain relapse. Running while performing some tactical or sport-specific task that requires attention, sudden change of direction or landing are situations related to an increased risk of an ankle sprain [1,3]. Multimodal comprehensive training protocols have been deemed as an effective therapeutic option to manage those with CAI [13,14]; nevertheless, the increased reliance on the visual information in CAI patients is not modified after this intervention [15,16]. The use of visual information at the expense of ankle joint somatosensory input has been identified as contributing factors for giving way episodes and repeated ankle sprains in those with CAI [17]. Conventional CAI interventions [15,16], do not address the necessity of restoring the altered somatosensorial deficits following an ankle sprain. Therefore, including a specific treatment option that focuses on this neurophysiologic dysfunction would be beneficial for these patients. Kim et al. [18] have reported the influence of stroboscopic vision (SV) to induce sensory reweighting of visual input in CAI patients. Stroboscopic vision consisted of the use of a special goggle whose glasses intermittently turn from transparent to opaque for 100 milliseconds and thus reduced the visual feedback. This kind of visual device has been previously employed for sporting purposes with positive results in baseball players [19], but until recently it has not been included in the rehabilitation process of patients with CAI. The implementation of this novel approach to CAI patients, may enhance the activity of the somatosensory pathways due to the limited visual information input, and contribute to the feedback–feedforward strategies’ development after training and its application to daily living and sporting activities.

The present study aimed to determine if the use of SV training in conjunction with traditional balance training will provide additional results in CAI patients.

## 2. Materials and Methods

This study was a single-blinded randomized controlled trial with two intervention groups consisting of multimodal balance training (BT), multimodal balance training in addition to stroboscopic glasses (BTSV), and a control group (CG) who received no intervention. The study (NCT04210518) was approved by the Human Ethics Committee of the University of Jaén and conducted following the Declaration of Helsinki, good clinical practices, and applicable laws and regulations and meets the CONSORT guidelines standards [20]. Informed consent was obtained for all participants who accepted to be enrolled in the study.

### 2.1. Participants and Randomization

Participants were recruited by posting announcements in the University newspaper, webpage, and by word of mouth. Participation eligibility was consistent with recommendations made from the International Ankle Consortium [21], determined by pre-screening measures including (1) a previous episode of ankle sprain at least 6 months before the beginning of the study, (2) a score of 24 or less in the Cumberland Ankle Instability Tool (CAIT) to confirm the current feeling of ankle joint instability, (3) no history of other musculoskeletal injuries in the lower limbs and, (4) mental and physical autonomy to participate in the intervention. Exclusion criteria for participants were (1) self-reported vestibular or balance-related dysfunction, (2) an acute ankle sprain within the last six weeks, (3) recent surgery, (4) epilepsy or a history of seizures, (5) participants were excluded if they missed more than 3 sessions. If patients presented bilateral CAI, the worst limb was selected as the CAI limb. Patients who met the inclusion criteria and accepted to be enrolled in the study were informed about the study protocol and advised to maintain their usual daily activity. To register any possible adverse events during the intervention, a notebook was given to the subjects of each group and permanent contact was maintained.

**Figure 1:**
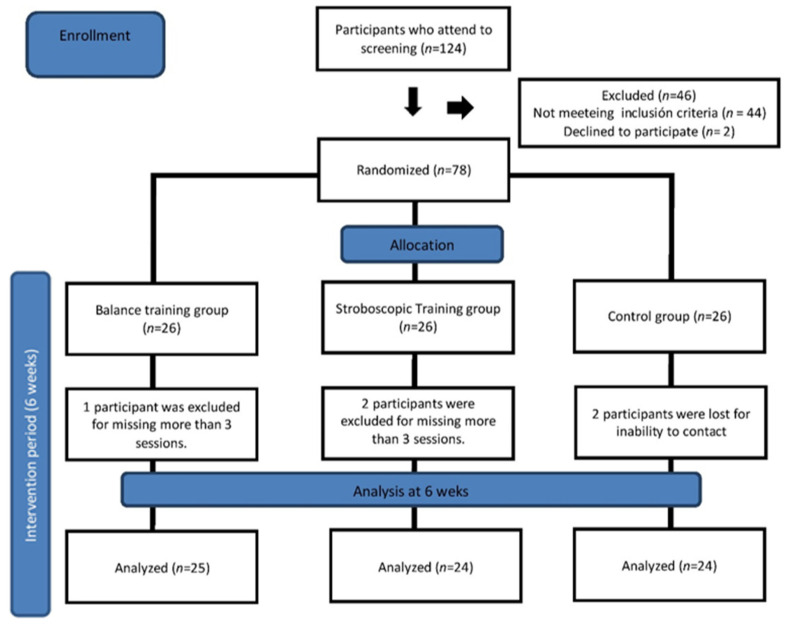
Flow chart of the study design and participants’ follow up through the trial.

A total of 78 participants were enrolled in the study, with 26 assigned to the BT, BTSV, and the CG. Five participants did not complete the study. Three participants missed more than three exercise sessions and two did not complete the study (Figure 1). The adherence rate in the intervention group was very high, with an overall 93.59% attendance. A total of 73 participants completed the study and were included for statistical analysis. Participant demographic information can be found in (Table 1).

An independent assessor who was blinded to group assignment completed all patients’ assessments at baseline and after the intervention.

A list of computer-generated numbers was employed to assign participants to the BT, BTSV, or CG. Participants were randomized to each group using sealed opaque envelopes that were controlled by an independent researcher who was not involved with the intervention in a 1:1:1 ratio before the beginning of the intervention.

### 2.2. Intervention

Intervention groups consisted of conventional balance training and conventional balance training in addition to SV.

### 2.3. Balance Training (BT)

This intervention consisted of a supervised multimodal exercise protocol addressing different aspects of balance including static and dynamic tasks on the injured ankle. The training program comprised 6 exercises that were progressively more difficult depending on the patient’s execution controlled by an expert physiotherapist (Table 2). The exercises were performed in a circuit format of 20 min of duration and all participants completed 18 training sessions divided into 6 weeks. Included exercises have been included in previous research [12,13,14] with positive findings and can be classified based on:

Single-limb stance. During these exercises, participants should maintain balance supported by the affected ankle. Progression consisted of the modification of the support surface (floor, mat, and dynair) and time (30” or 60”).

Throwing and catching task. During this station, patients should combine a single-limb stance on the affected ankle with throwing and catching drills. The difficulty of these exercises consisted of the modification of the support surface making it more unstable (floor, mat, dynair, and bosu). The objective of this exercise was to avoid attentional focus on ankle stability simulating sports activities that require attention to an external stimulus.

Single-leg deadlift. This exercise was included to enhance the coordination and strength of the lower limb. Progression consisted of limiting the use of the arms to maintain balance, adding some weight during the execution, and finally reaching three points placed in front of the patient in a more dynamic task.

Single-limb hops to stabilization. Instability and residual symptoms after landing from a hop, as well as lateral displacements, have been reported in those with CAI. Previous research has addressed this CAI contributing factor with similar interventions based on the multidirectional jump protocol [12,13,14]. Progression consisted of an increase in the jump distance.

The balance training protocol was performed in an indoor sports facility with no shoes and in a small group.

### 2.4. Balance Training and Stroboscopic Vision (BTSV)

Patients included in this group performed the same protocol described in the balance training group with the addition of the SV glasses (USD 299, Senaptec LLC, Beaverton, OR, USA) during the intervention. Participants wear SV glasses all time alternating between opaque and transparent phase with a total transparent mode during rest. Visual difficulty progression ranged from 1 to 8 levels and was individually determined and adjusted by the correct exercise execution during the 18 training sessions. The opaque/transparent ratio can be easily controlled by the lateral buttons placed on the glasses. Participants were advised to participate in the training session with their usual glasses or contact lenses. The training session was stopped if dizziness or any adverse event was observed during the exercise performance.

### 2.5. Control Group

Participants in the control group received no intervention.

### 2.6. Outcome Measures

#### 2.6.1. Primary Outcome Measures

Ankle dorsiflexion range of motion: DFROM was assessed by the weight-bearing lunge test (WBLT) which has been shown to have high inter-rater (r = 0.99) and interrater (r = 0.98) reliability [22]. Furthermore, it has been applied to patients with CAI, where it has proven a correlation with dynamic postural control measures [23].

Dynamic balance: Dynamic balance was measured by a simplified version of the Star Excursion Balance Test (SEBT) where the anterior, posteromedial, and posterolateral reach directions were assessed and have been reported to determine dynamic postural control [20,21]. SEBT has been deemed to be a reliable and valid test to detect reach deficits both between subjects and within sides of subjects with unilateral ankle instability [24,25,26].

#### 2.6.2. Secondary Outcome Measures

Self-reported instability and function: To determine the severity of ankle instability the Cumberland ankle instability tool (CAIT) was used [27]. The CAIT has been reported to be a valid tool with discriminative properties to identify patients with CAI and scores ≤ 25 are considered as unstable ankles [28,29].

Functional status: Function in daily living and sports activities were assessed by the Foot and Ankle Ability Measure (FAAM). The FAAM is a two-dimension (sport and daily life activities) non-specific disease questionnaire that has been reported to detect self-reported functional deficits in CAI patients [30,31].

### 2.7. Statistical Analyses

The Chi-Square test was applied to detect significant differences in the case of categorical variables. In the rest of the variables, to detect significant differences among the three groups, the appropriate technique is the Analysis of variance (ANOVA). For the conclusions to be valid in this technique, it is necessary to verify a series of assumptions. The residuals of the model have to fulfill the following hypotheses: normality, randomness, independence, and homoscedasticity.

The Shapiro–Wilk test was used to test the normality hypothesis and the Levene test was used to test the homoscedasticity hypothesis. In those cases, in which one of the two hypotheses is not verified, the ANOVA technique cannot be applied, since the results would not be reliable. In these cases, the ANOVA technique was replaced by the Kruskal–Wallis test. In cases that verify these two hypotheses, the ANOVA technique was applied. On the other hand, when ANOVA or the Kruskal–Wallis test is significant (*p*-value < 0.05) it indicates that there are significant differences between at least a couple of groups. To determine exactly between which groups there are significant differences, we completed the study with the methods of multiple comparisons or post hoc comparisons. We used two post hoc techniques: in cases where the homoscedasticity hypothesis was verified, HDS Tukey test was applied. In cases where it was not verified, the Games–Howell test was applied (because this technique is more suitable when the homoscedasticity hypothesis is not verified). Furthermore, to carry out the study WITHIN-GROUP CHANGE SCORES, confidence intervals were calculated for the difference in means for paired or dependent samples, hypothesis tests for two dependent or paired samples and Cohen’s D.

Alpha level was set a prior at *p* < 0.05. Statistical analysis was performed using the SPSS software, version 20.0 (SPSS Inc., Chicago, IL, USA). The α level was set at *p* < 0.05 for all tests.

A required number of 21 participants with CAI per group was estimated to ensure a power of 0.80 at a significance level of 95% based on a minimal detectable change of 4.28 cm in the posterolateral direction of SEBT [9], but 26 participants per group were enrolled in the study due to a possible drop-out rate of 20%.

## 3. Results

In the baseline analysis the mean and the standard deviation (continuous variables) and the absolute and relative frequency (categorical variables) are shown for each variable, for each group, and the total number of individuals. Both groups were similar at baseline for all dependent and sociodemographic variables (Table 1 and Table 3).

**Figure 2:**
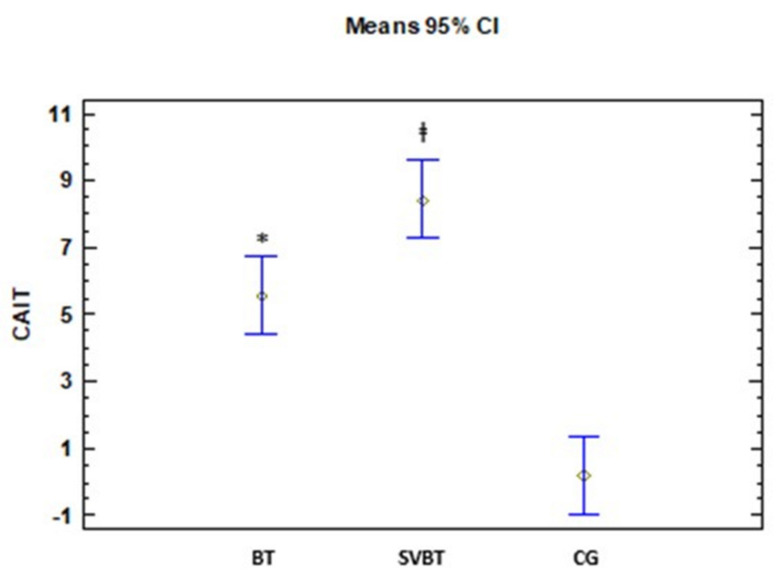
CAIT change scores after the intervention. Cumberland ankle instability tool (CAIT). ‡ (SVBT > BT); * (BT > CG).

**Figure 3:**
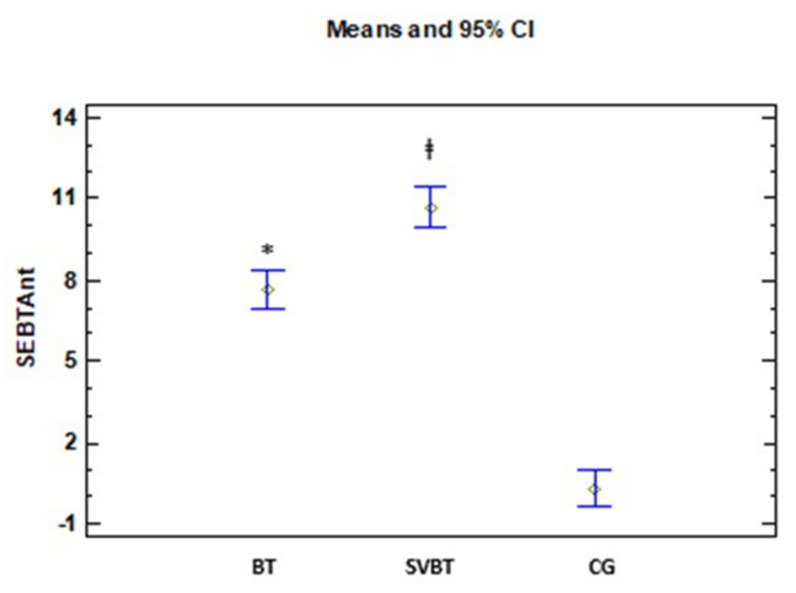
Anterior SEBT change scores after the intervention. Anterior reach distance of the star excursion balance test (SVBT any). ‡ (SVBT > BT) * (BT > CG).

In both BT group and group BTSV, there are significant differences in the mean of all variables comparing the values at the beginning and end of the study, so that the value of the mean end of the study is greater than at the beginning in all the variables. In contrast, in group 3 (control), there are no significant differences between the means at the beginning and end of the study (Table 3). On the other hand, in this same table, in all the variables there are significant differences in the BT and BTSV groups with the CG, in such a way that the means of these variables in those groups are greater than in the CG. Regarding the obtained results in the variables SEBT-Ant and CAIT, there are also significant differences between BT and BTSV, with μ1 < μ2 (Figure 2 and Figure 3).

## 4. Discussion

The present study provides preliminary evidence, based on the PEDro score [32] of 8 out of 10 possible points, that incorporating stroboscopic vision (SV) into balance training (BTSV) was found to be more effective than balance training (BT) alone in patients with CAI, in the short term of this 6-week trial.

Both training groups showed significant improvements in all outcomes (i.e., CAIT, FAAM, SEBT, and DFROM) when compared with the CG not receiving any intervention. These training effects were found to be very large for all outcomes (d = 1.52 to 7.79). More importantly, there were further improvements in the BTSV compared with the BT: a large effect for SEBT-Ant. (d = 1.2, 95% CI = −4.8 to −1.27) and a close to large effect for CAIT (d = 0.72, 95% CI = −5.62 to −0.08) with their associated 95% CIs not crossing zero. These additional improvements with the addition of SV to BT are unique and may assist clinicians in improving their current rehabilitation strategies for CAI patients.

### 4.1. Patient-Reported Outcomes

The most important finding of the current study was the additional improvement in the CAIT score following BTSV. CAIT is an instrument that provides a cut-off score (i.e., <25 pts) to confirm self-reported ankle instability, and its score is often used to determine a level of perceived ankle instability, with a lower score indicating worse instability [27,33]. For these reasons, assessment of the severity of perceived instability with CAIT has been increasingly used in rehabilitation for CAI [7,9,29]. Previous research has shown that balance training consistently produces large effects on CAIT scores in CAI patients: Cruz-Diaz et al. [9], (d = 2.12, 95% CI = 1.54–2.71), Kim and Heo [33] (d = 1.22, 95% CI = 0.27–2.18), Wright et al. [7] (d = 1.17, 95% CI = 0.50–1.84). We also confirmed the large (pre-post change) effect (d = 1.19) found in the group receiving BT alone. Moreover, the large improvement may be a clinically meaningful change as the size of improvement (5.56 pts) exceeds its established Minimum Clinically Important Difference (MCID) value (3 pts) [34]. Nonetheless, balance training alone may not be sufficient as the addition of SV to BT further decreased perceived ankle instability, as indicated by the moderate difference (d = 0.72) between the BT and BTSV in pre-post change scores. This finding is unique because visual perturbation during balance training by imitating visual input by half with SV may further tune the postural control system and make participants with CAI feel less ankle instability. Despite the promising effect of SV on perceived ankle instability, further research is warranted to determine whether this perceptual effect is related to a decrease in the number of episodes of the ankle giving way or subsequent ankle injuries.

In contrast to additional improvement in the CAIT score following BTSV, we did not observe any significant differences between the training groups for perceived ankle function, as quantified by FAAM scores. However, both training groups significantly increased their FAAM-Sport scores (BT pre-post change: 16.4 pts, BTSV pre-post change: 17.9 pts) and the training effects were large (BT: d = 2.64, BTSV: d = 2.60) and greater than its established MCID value (9 pts) [35]. Similarly, both training groups produced significant large effects on the FAAM-ADL score, but the improvement following BT alone was not greater than its MCID value (8 pts) while BTSV induced a training effect greater than the MCID [7]. These results indicate that BT alone is adequate in making clinical meaningful changes in perceived ankle function during sporting activities, but it may not be for activities of daily living, which may benefit from additional therapy like SV. We found additional moderate improvement (d = 0.56) in the FAAM-ADL score in the BTSV compared with BT, but the group difference was not conclusive because its 95% CI (−9.82 to 1.05) crossed zero.

### 4.2. Dynamic Balance

We found both training groups had large effects on reach distances of SEBT in all directions. These improvements appear to be consistent with previous BT studies [7,36,37,38,39]. Interestingly, the current study found that the incorporation of SV into BT induced an additional large effect (d = 1.2, 95% CI = −4.8, −1.27) on the anterior reach distance compared with BT alone. In contrast, we did not find significant SV effects in other directions.

### 4.3. Dorsiflexion Range of Motion

Dorsiflexion range of motion (DFROM), assessed by the weight-bearing lunge test, was not significantly different between the BTSV and BT groups: the addition of SV to BT did not induce any additional improvement. However, we found large improvements in both training groups compared with the CG (BT: d = 1.87, BTSV: d = 1.52). This mechanical benefit associated with BT may be attributed to dynamic postural control components of BT (i.e., reaching and hopping) in these studies. Previous research established a link between DFROM and dynamic balance in CAI patients [23,40,41]. It is plausible that progressive dynamic activities during BT in the current study required increased ankle movements and resulted in DFROM improvement. From this perspective, BT is versatile for improving DFROM while addressing other sensorimotor (or patient-reported) deficits [42,43,44]. Nonetheless, a more direct mechanical intervention such as joint mobilization cannot be ruled out when considering an intervention for DFROM deficits in CAI patients. A recent meta-analysis [45] produced grade A evidence that ankle joint mobilizations are effective in improving DFROM and recommended Maitland mobilization or Mulligan mobilization with movement that targets the posterior glide of the talus and manipulations of the talocrural joint. Thus, clinicians more concerned with DFROM deficits in CAI patients should consider this recommendation.

### 4.4. Potential Neurophysiological Mechanisms

As the current study was to determine the efficacy of BTSV for CAI patients, it does not provide data regarding the underlying neurophysiological mechanism responsible for additional improvements found in the BTSV. However, previous research suggests that improvements in physical performance following exercise training with SV may be due to increased utilization of sensory input other than the visual one and/or improved efficiency of visual–motor processing [46,47]. From these two mechanistic perspectives, the additional training effects with SV found in the current study may be explained. First, participants during BT with limited visual input (SV) were forced to utilize more sensory feedback from other sources such as the somatosensory system. This inter-modality sensory reweighting from the visual to somatosensory systems may be required for participants to accommodate postural demand arising from balance exercises, which would increase the somatosensory load and induce greater training effects [18,40]. Previous studies [48,49,50,51] reported similar findings that exercise training with SV induces additional perceptional and physical improvements including balance. However, none of the studies have directly addressed this potential mechanism. Another potential mechanism may be improved visual–motor processing [48]. Some of our balance exercises were performed with catching/throwing a ball or reaching. These additional activities require visual resources, and participants performing these balance exercises under SV would have to utilize limited visual input more efficiently, which improves temporal integration of sensory information and induces greater motor improvements [48]. However, specific mechanisms remain unclear while various visual–motor skill improvements are often found to relate to better physical performance. In particular, the latest study [51] examined a possible sensory mechanism with electroencephalography in top-level badminton players, with two groups: SV training and control groups, both of which underwent a 4-week badminton training. The SV training group showed better performance (i.e., smash-defense tests), but the group difference in visual processing speed (i.e., N2 latency) did not reach significance although higher performance gains following training were associated with faster visual processing. Authors attributed this insignificant result to their small sample size (*n* = 10), training-induced neurophysiologic adaptions that may occur in motor systems, and/or improved attentional resources [51]. The importance of visual input on postural control has been addressed by previous studies [52] and might be consider during the therapeutic approach of the studied population group.

### 4.5. Clinical Implications

The current study provides evidence that the use of SV may be beneficial in CAI rehabilitation, particularly in a functional stage of rehabilitation when a CAI patient is preparing to return to play or work. Therapeutic exercises are often prescribed in these patients in a controlled environment, where the primary attention is on the success of exercise performance (control of body mechanics). Exercises are beneficial in an early stage of rehabilitation (i.e., prevention of muscle atrophy, recovery of neuromuscular control and muscle function), but in a later (functional) stage of rehabilitation, they may not be challenging enough to represent various demands placed on patients during daily activities of living and sports [53]. These activities often require multiple tasks, beyond the simple task (i.e., exercise performance in a rehabilitation setting), which makes the primary attentional resources shift from sensorimotor control of exercise to other higher-level tasks (i.e., going across the street, playing with others, etc.). SV may provide a more challenging environment where participants have to efficiently utilize limited visual input to perform their assigned rehabilitation exercises, which could further improve their sensorimotor control, including postural control.

In addition, improvements following therapeutic exercise are often task-specific: improvement is obtained in a task that has been practiced [54,55]. For this reason, SV may be even more beneficial for athletic patients because it can be applied to their regular sport-specific training to promote the full recovery of sports performance. Furthermore, SV may enhance patient compliance with sports rehabilitation. Athletic patients often feel bored when performing a therapeutic exercise, resulting in a low compliance rate [56]. However, athletes who underwent SV training reported that training with SV was enjoyable and highly motivating, and it is suggested that training with SV can be implemented with minimal guidance such as a home exercise program [49]. Similar to other therapeutic agents, however, SV has contradictions such as epilepsy or a history of seizures [46]. Sports medicine practitioners should be aware of these conditions when considering incorporating SV into their exercise training/rehabilitation.

### 4.6. Study Limitations and Recommendations for Future Research

While the current study shows promising results for use of SV in CAI patients, we acknowledge that placebo effects may happen in the BTSV group. It has been the greatest challenge in the SV training literature in that it may be impossible to blind either participants or therapists to the visual experience [46]. One would argue that a placebo condition can be made with constant SV (the frequency of visual blocks is fixed at the easiest setting) as opposed to the active training condition with variable SV (the frequency varies). While this idea sounds very appealing, a recent study [49] examined these two SV conditions and reported no differences in visual and physical function. Some authors suggest that the mere interruption of visual input, regardless of whether it is constant or variable, is strong enough to induce expected effects [57].

The study findings are limited to 6-week trial effects. It is unknown whether the SV training effects found in the current study may last or disappear in the long term (i.e., 6 months) and how some factors such as age, gender, or limb dominance could influence the studied variables. Further studies assessing long-term effects and the application on different injuries may demonstrate the efficacy of SV training. Identifying dominant vs. non-dominant at baseline could be beneficial to determine the difference in the effectiveness of the intervention over time.

## 5. Conclusions

The current study provides preliminary and short-term findings to demonstrate that CAI patients may benefit from a visually challenging rehabilitation environment, created by SV (rapid and repetitive visual interruption). Incorporating SV into BT showed some improvements (large effects) in CAI patients regarding perceived ankle instability and dynamic postural control (i.e., anterior reach during SEBT) relative to BT alone while both (BTSV and BT alone) interventions produced large therapeutic effects in all outcomes (i.e., CAIT, FAAM, SEBT, and DFROM).

## Figures and Tables

**Table 1:** Sociodemographic characteristics at baseline.

Characteristics		Balance Training (*n* = 25)	Stroboscopic Vision (*n* = 24)	Control (*n* = 24)	*p*-Value
Age (years)		29.76 ± 10.009	27.38 ± 7.383	29.67 ± 9.407	0.755
Height (cm)		167.48 ± 22.946	170.63 ± 8.791	170.50 ± 9.758	0.963
Mass (kg)		73.22 ± 21.122	71.94 ± 9.891	69.38 ± 9.188	0.374
Gender	Male	12/48%	17/70.83%	13/54.17%	0.249
	Female	13/52%	7/29.17%	11/45.83%	
Occupational status					0.724
	Full-Time worker	18/72%	16/66.6%	19/79.16%	
	Part-time worker	2/8%	1/4.16%	2/8.33%	
	unemployed	5/20%	7/29.16%	3/12.5%	
Education					0.912
	Primary	4/16%	5/20.83%	4/16.6%	
	Secondary	8/32%	10/41.26%	11/45.83%	
	University	13/52%	9/37.5%	9/37.5%	
Affected ankle	Left	12/48%	9/37.5%	11/45.83%	0.738
	Right	13/52%	15/62.5%	13/54.17%	
DFROM		8.348 ± 2.854	6.733 ± 2.886	8.821 ± 0.8876	0.317
SEBT-Ant		78.016 ± 3.519	77.337 ± 4.748	74.929 ± 2.8403	0.124
SEBT-PM		85.524 ± 4.350	83.238 ± 5.045	83.208 ± 4.4112	0.271
SEBT-PL		84.252 ± 4.823	81.871 ± 6.884	80.946 ± 4.7340	0.089
CAIT		15.28 ± 4.650	15.13 ± 4.803	15.29 ± 5.645	0.934
FAAM-ADL		79.60 ± 6.278	74.04 ± 8.175	72.50 ± 9.325	0.067
FAAM-Sport		69.60 ± 6.278	69.17 ± 6.197	67.29 ± 6.252	0.232

Ankle dorsiflexion range of motion (DFROM); Anterior reach distance of the star excursion balance test (SEBT-Ant); Posteromedial reach distance of the star excursion balance test (SEBT-PM); Posterolateral reach distance of the star excursion balance test (SEBT-PL); Cumberland ankle instability tool (CAIT); Functional ankle ability measure daily living subscale (FAAM-ADL); Functional ankle ability measure sport subscale (FAAM-Sport).

**Table 2:** Intervention protocol and progression.

	Progression 1	Progression 2	Progression 3
Crossed arms, single-limb stance on the floor (60”)	Crossed arms, single-limb stance on a mat (30”)	Crossed arms, single-limb stance on a mat (60”)	Crossed arms, single-limb stance on a dynair (30”)
Throwing/catching a ball. single-limb on the floor (10 repetitions)	Throwing/catching a ball. Single-limb on a mat (10 repetitions)	Throwing/catching a ball. Single-limb on a dynair (10 repetitions)	Throwing/catching a ball. Single-limb on a bosu (10 repetitions)
Single leg deadlift with open arms (10 repetitions)	Single leg deadlift with hands at the hip (10 repetitions)	Single leg deadlift with a lightweight (10 repetitions)	Single leg deadlift reaching 3 points (10 repetitions)
Lateral hop to stabilization hands at the hip (10 repetitions)	Lateral hop to stabilization with hands at the hip (10 repetitions) (30 cm)	Lateral hop to stabilization with hands at the hip (10 repetitions) (45 cm)	Lateral hop to stabilization with hands at the hip (10 repetitions) (1 m)
Back and forward hop to stabilization with hands at the hip (10 repetitions)	Back and forward hop to stabilization with hands at the hip (10 repetitions) (30 cm)	Back and forward hop to stabilization with hands at the hip (10 repetitions) (45 cm)	Back and forward hop to stabilization with hands at the hip (10 repetitions) (1 m)
Randomized hop to stabilization in 4 directions (10 repetitions)	Increasing the speed during the performance (physiotherapist randomly indicates the direction).		
Circuit was repeated two times with 30” of rest between exercises and 2′ of rest between each circuit.			

**Table 3:** Within-group and between-group change scores.

			Within-Group Change Scores			Between-Group Differences in Change Scores		
Variable	Baseline Mean ± SD	End of Treatment Mean ± SD	Mean (95% CI)	*p*-Value	d-Cohen	Mean (95% CI)	*p*-Value	d-Cohen
DFROM								
Games–Howell		Kruskal–Wallis → *p* = 0.000						
BT	8.348 ± 2.854	10.016 ± 2.637	−1.6680 (−2.1876, −1.1484)	0.000	0.607	BT-BTSV: −0.302 (−1.3995, 0.793)	0.781	BT-BTSV: 0.192
BTSV	6.733 ± 2.886	8.704 ± 2.927	−1.9708 (−2.7444, −1.1973)	0.000	0.678	BT-CG: 1.681 (1.046, 2.314)	0.000	BT-CG: 1.869
CG	8.821 ± 0.887	8.808 ± 0.876	0.0125 (−0.0625, 0.0875)	0.734	0.014	BTSV-CG: 1.983 (1.043, 2.923)	0.000	BTSV-CG: 1.523
SEBT-Ant								
Games–Howell		Kruskal–Wallis → *p* = 0.000						
BT	78.016± 3.519	85.668± 3.481	−7.6520 (−8.0262, −7.2778)	0.000	2.185	BT-BTSV: −3.039 (−4.801, −1.278)	0.001	BT-BTSV: 1.235
BTSV	77.337 ± 4.748	88.029 ± 3.979	−10.6917 (−12.1100, −9.2734)	0.000	2.440	BT-CG: 7.335 (6.683, 7.987)	0.000	BT-CG: 7.787
CG	74.929 ± 2.840	75.246 ± 2.822	−0.3167 (−0.7288, 0.0955)	0.126	0.112	BTSV-CG: 10.375 (8.604, 12.145)	0.000	BTSV-CG: 4.194
SEBT-PM								
Games–Howell		Kruskal–Wallis → *p* = 0.000						
BT	85.524 ± 4.350	92.228 ± 3.570	−6.7040 (−8.0703, −5.3377)	0.000	1.684	BT-BTSV: −2.533 (−6.245, 1.178)	0.230	BT-BTSV: 0.481
BTSV	83.238 ± 5.045	92.475 ± 4.965	−9.2375 (−12.0535, −6.4215)	0.000	1.845	BT-CG: 6.199 (3.738, 8.661)	0.000	BT-CG: 1.746
CG	83.208 ± 4.411	83.713 ± 6.131	−0.5042 (−2.0985, 1.0901)	0.519	0.094	BTSV-CG: 8.733 (4.911, 12.555)	0.000	BTSV-CG: 1.611
SEBT-PL								
Games–Howell		Kruskal–Wallis → *p* = 0.000						
BT	84.252 ± 4.823	90.708 ± 4.460	−6.4560 (−8.4358, −4.4762)	0.000	1.389	BT-BTSV: −1.960(−5.708, 1.787)	0.420	BT-BTSV: 0.363
BTSV	81.871 ± 6.884	90.288 ± 2.958	−8.4167 (−10.9235, −5.9099)	0.000	1.588	BT-CG: 5.514 (2.886, 8.142)	0.000	BT-CG: 1.457
CG	80.946 ± 4.734	81.888 ± 4.238	−0.9417 (−1.9420, 0.0587)	0.064	0.209	BTSV-CG: 7.475 (4.259, 10.690)	0.000	BTSV-CG: 1.653
CAIT								
Games–Howell		Kruskal–Wallis → *p* = 0.000						
BT	15.282 ± 4.650	20.840 ± 4.696	−5.560 (−6.898, −4.222)	0.000	1.189	BT-BTSV: −2.856 (−5.629, −0.084)	0.042	BT-BTSV: 0.718
BTSV	15.137 ± 4.803	23.541 ± 2.843	−8.417 (−10.357, −6.476)	0.000	2.130	BT-CG: 5.351 (3.557, 7.146)	0.000	BT-CG: 2.069
CG	15.290 ± 5.645	15.503 ± 4.987	−0.208 (−0.923, 0.507)	0.553	0.039	BTSV-CG: 8.208 (5.739, 10.676)	0.000	BTSV-CG: 2.370
FAAM-ADL								
Games–Howell		Kruskal–Wallis → *p* = 0.000						
BT	79.602 ± 6.278	86.800 ± 8.021	−7.200 (−9.935, −4.465)	0.000	0.999	BT-BTSV: −4.383 (−9.826, 1.059)	0.136	BT-BTSV: 0.560
BTSV	74.044 ± 8.175	85.630 ± 5.578	−11.583 (−15.323, −7.844)	0.000	1.656	BT-CG: 6.783 (3.087, 10.479)	0.000	BT-CG: 1.273
CG	72.500 ± 9.325	72.920 ± 9.315	−0.417 (−1.931, 1.098)	0.575	0.045	BTSV-CG: 11.166 (6.361, 15.971)	0.000	BTSV-CG: 1.652
FAAM-sport								
Games–Howell		Kruskal–Wallis → *p* = 0.000						
BT	69.602 ± 6.278	86.000 ± 6.124	−16.400 (−18.508, −14.292)	0.000	2.644	BT-BTSV: −1.5166(−6.468, 3.434)	0.737	BT-BTSV: 0.214
BTSV	69.174 ± 6.197	87.080 ± 7.506	−17.917 (−21.543, −14.291)	0.000	2.602	BT-CG: 15.983 (12.933, 19.033)	0.000	BT-CG: 3.622
CG	67.290 ± 6.252	67.710 ± 6.252	−0.417 (−1.931, 1.098)	0.575	0.067	BTSV-CG: 17.500 (12.823, 22.176)	0.000	BTSV-CG: 2.659

Balance training group (BT); Stroboscopic vision training group (BTSV); Control group (CG); Ankle dorsiflexion range of motion DFROM; Anterior reach distance of the star excursion balance test (SEBT-Ant); Posteromedial reach distance of the star excursion balance test (SEBT-PM); Posterolateral reach distance of the star excursion balance test (SEBT-PL); Cumberland ankle instability tool (CAIT); Functional ankle ability measure daily living subscale (FAAM-ADL); Functional ankle ability measure sport subscale (FAAM-Sport).

## Data Availability

Not applicable.
